# Occipital event-related potentials to addiction-related stimuli in detoxified patients with alcohol dependence, and their association with three-month relapse

**DOI:** 10.1186/s12888-016-0782-0

**Published:** 2016-03-21

**Authors:** Carolin Matheus-Roth, Ingmar Schenk, Jens Wiltfang, Norbert Scherbaum, Bernhard W. Müller

**Affiliations:** Department for Addiction Medicine and Addictive Behavior, LVR-Hospital Essen, Faculty of Medicine, University of Duisburg-Essen, Virchowstrasse 174, 45147 Essen, Germany; Rehaklinik Bellikon, Mutschellenstrasse 2, CH-5454 Bellikon, Switzerland; Department of Psychiatry and Psychotherapy, University Medical Center (UMG), Georg-August-University, Von-Siebold-Str. 5, 37075 Göttingen, Germany; Department for Psychiatry and Psychotherapy, LVR-Hospital Essen, Faculty of Medicine, University of Duisburg-Essen, Virchowstrasse 174, 45147 Essen, Germany; Department of Psychology, University of Wuppertal, Gaußstraße 2 0, 42119 Wuppertal, Germany

**Keywords:** Alcohol addiction, Relapse, Cue-reactivity, Event-related potentials

## Abstract

**Background:**

Understanding the biological underpinnings of relapse in alcohol dependency is a major issue in addiction research. Based on recent evidence regarding the relevance of occipital visual evoked response potentials (ERPs) in addiction research, and its significance for relapse research, we assessed occipital ERPs to alcohol- and non-alcohol-related stimuli in recently detoxified patients and controls.

**Methods:**

Thirty recently detoxified patients with alcohol addiction, and 31 healthy control subjects, were assessed in a Go and a NoGo condition, each using three visual stimuli: tea, juice and beer. In the “Go” condition, subjects had to respond to the juice (12.5 %) and the beer stimulus (12.5 %), and ignore the tea picture (75 %). In the “NoGo” condition, subjects had to respond to the tea picture (75 %) and ignore the juice and the beer picture (12.5 % each). The subjects’ EEGs were analyzed with regard to the occipital P100 and N170 ERP components. Patients were then evaluated for relapse 3 months after this initial assessment.

**Results:**

P100 amplitudes differed between conditions and between stimuli, and we found a condition x electrode interaction. However, none of these P100 results involved group or relapse-status effects. N170 amplitudes in patients were elevated as compared to controls. Additionally, patients’ heightened N170 amplitudes in response to the alcohol-related (beer) stimulus were found only under the NoGo condition, where subjects had to react to the frequent tea stimulus and ignore the beer and the juice stimuli, thus resulting in a condition x stimulus x group interaction. Patients reporting relapse in a 3-month follow-up assessment showed larger NoGo N170 alcohol cue-related ERP amplitudes and increased depression scores as compared to patients who stayed abstinent. Depression was related to shortened P100 latencies in patients, but unrelated to the N170 NoGo cue-reactivity effect.

**Conclusions:**

Our results indicate a sensitivity of occipital ERPs to addiction-related stimuli when these act as non-targets. Recently detoxified patients may be vulnerable to addiction-related cues when these occur outside the focus of directed attention, thereby circumventing intentional control processes. Furthermore, ERPs to addiction-related stimuli may be useful as a predictor of abstinence success in recently detoxified patients.

**Electronic supplementary material:**

The online version of this article (doi:10.1186/s12888-016-0782-0) contains supplementary material, which is available to authorized users.

## Background

Alcohol use is among the most relevant risk factors contributing to disease-related death and disability-adjusted life years worldwide [1], with relapse prediction being a major issue in the treatment of alcohol use disorder [2, 3].

Investigations of brain responses to addiction-related cues have emerged as a door to the assessment of relapse behavior; and the incentive salience theory [4] has been established as a major contribution providing a theoretical framework for research into cue reactivity. A major suggestion of this theory is that not only is the drug associated with increased and persistent incentive salience, but that drug-related cues and even drug-related context signals elicit biased attention, trigger dopamine-related reward systems and may lead to active drug approach behavior, thus initiating relapse [5].

Studies on cue reactivity have measured altered peripheral physiological signals [6], evoked response potential (ERP) data derived from EEGs [7, 8] and neuroimaging brain activation changes [9] to drug- related cues. Carter and Tiffany (1999), for example, analyzed more than 40 studies measuring cue- related changes in physiological signals [6]. With regard to alcohol addiction, they reported a medium effect size of d = .53 for the induction of craving, and of d = .39 for increases in heart rate with exposure to addiction-related stimuli. With regard to ERP research, Littel et al. [7] reviewed studies which focused on slow wave potentials and the P300 ERP component, which are usually measured at the frontal to parietal electrode sites. Patients with substance abuse disorders showed heightened P300 and slow wave amplitudes in reaction to drug-related stimuli [10–12]. These results are of importance since exaggerated ERP amplitudes to drug cues have been associated with relapse behavior. For example, Petit and colleagues [12] recently found that P300 oddball task parameters in response to alcohol stimuli are related to successful 3-month abstinence in detoxified alcohol dependent patients. In this study, 3 month successful alcohol abstinence was associated with reduced P300 amplitudes in response to addiction-related stimuli as compared to patients relapsing within the 3 month evaluation period, indicating the relevance of evoked potential measures as probable indicators of treatment success.

Expanding upon this more traditional approach to ERP research in alcohol use disorders, Maurage and colleagues established the assessment of occipital evoked potential components which allows the evaluation of early visual processing in research on alcohol use disorders [13–15]. The occipital positive P100 component indexes early visual processing, and its amplitude is more closely confined to the perceptual properties of the stimulus as compared to the negative N170 component, which has been widely studied with regard to face processing [16, 17]. In a recent meta-analysis on studies assessing the N170 in the context of face processing, the authors not only found that this component is sensitive to emotional expressions, but also that the N170 was modulated in amplitude when the attention of subjects was directed to non-face-related visual target features [18]. In another study by Hietanen & Nummenmaa [19], authors reported evidence that affective arousal elicited by naked body stimuli significantly enhanced N170 amplitudes, and Tanaka & Curran [20] reported differentially enhanced N170 amplitudes in dog and cat experts when shown stimuli related to their area of expertise, indicating that non-face stimuli may contribute to the amplitudes of the occipital N170 component.

In line with the research focus on the N170 component in face processing experiments, Maurage [14] assessed emotional face processing and reported prolonged P100 and N170 latencies, and lower N170 amplitudes, in recently detoxified alcohol-dependent patients. Early visual processing abnormalities with respect to face stimuli were replicated in alcohol dependent patients and in binge drinkers [13, 21]. Although these studies targeted aspects of visual face processing in general, and not addiction-specific cue reactivity, they demonstrated that it is worthwhile to study occipital processing in experiments on stimulus processing in addicted individuals. This is further supported by a recent study by Petit et al. [15] reporting increased occipital P100 amplitudes in binge drinkers in response to rare alcohol-related target stimuli as compared to non-alcohol-related target stimuli, indicating that these stimuli influence the very early stages of cortical processing in subjects prone to develop alcohol addiction. Taken together, these studies support the idea that, with respect to the occipital P100 and N170 components, alcohol-related stimuli may be processed differently from non-alcohol-related stimuli, when they act as targets. Due to their probable specific significance for addicted patients, and in accordance with the results of Hinojosa et al. [18] and Hietanen et al. [19] on non-face stimulus characteristics affecting the N170 component, this may even occur when drug related stimuli are presented as non-target stimuli.

In the present study, we aimed to investigate the P100 and N170 components of early occipital visual processing in response to alcohol and non-alcohol-related pictures in recently detoxified alcohol dependent patients. We applied a conventional oddball paradigm using a frequently presented non-alcohol-related and non-target stimulus (tea) in combination with non-alcohol (orange juice) and alcohol-related target stimuli (beer, Go-condition). In a second part of the experiment, the tea stimulus acted as a target, and the same two less frequently presented stimuli acted as to-be-ignored non-target stimuli (NoGo condition). In addition, we examined the relation between these parameters and patients’ 3-month relapse status. Based on existing evidence, and given the specific significance of alcohol-related stimuli to alcohol-dependent patients, we expected heightened occipital P100 and N170 amplitudes in patients, but not controls, in response to drug-related stimuli, specifically when such stimuli act as targets, and perhaps even when attention is focused on non-drug stimuli. Successful 3-month abstinence may be associated with lowered ERP amplitudes when taking into consideration the results of Petit et al. [12].

## Methods

### Subjects

We assessed 30 patients with alcohol dependence and 31 control subjects. All subjects gave written informed consent before participation in the study. The study was approved by the ethics committee of the faculty of medicine of the University of Duisburg-Essen.

Participating patients had recently successfully completed an inpatient detoxification treatment program in a specialized hospital ward for addictive disorders. Symptoms of alcohol withdrawal were alleviated by temporary administration of clonazepam or clomethiazole, respectively, according to the German treatment guidelines regarding the detoxification of alcohol addicts [22]. Medication dosage was based on the monitoring of withdrawal intensity by a symptom checklist.

The inclusion criteria for *patients* were alcohol dependency (DSM-IV 303.9), and age (between 18 and 60 years). Exclusion criteria were use of psychotropic medication and sedatives (e.g. benzodiazepines, antidepressant medication) other than zopiclone, a history or presence of substance abuse or dependency other than alcohol (apart from nicotine), uncorrected vision problems, and major psychiatric axis-I co-morbidity (e.g. schizophrenia, major depression). The one inclusion criterion for controls was age (between 18 and 60 years). Exclusion criteria were present or past substance dependency (apart from nicotine), major psychiatric disorders (e.g. schizophrenia or major depression), any psychotropic medication, and uncorrected vision problems. Controls received 40 Euros in exchange for their participation in the study. Controls were recruited through local advertisement and were matched for patients’ age, gender and level of education Table 1.

**Table 1:** Subject characteristics: patients and control subjects

	Control (*n* = 31)	Patient (*n* = 30)	p =
Age y (std)	43.3 (9.0)	44.3 (8.0)	.56 (ANOVA)
Handedness (n, right)	29	29	.57 (Chi^2^)
Gender (n: f/m)	6/25	5/25	.79 (Chi^2^)
Nicotine dependency (n)	16	23	.02 (Chi^2^)
Fagerström Score mean (sd)	2.55 (3.0)	5.63 (3.9)	.001(ANOVA)
School (n:without/low/mid/high*):	0,9,10,12	1,14,6,9	.32 (Chi^2^)
Preference drink (n:beer/wine/other)	19/6/5	20/6/4	.85 (Chi^2^)
Health-related problems (n)	1.48 (5.60)	9.00 (13.14)	.005 (ANOVA)
Breath alcohol concentration in per mille (sd)**	-	1.15 (1.30)	-
AUDIT mean (std)	2.71 (1.83)	24.50 (7.71)	<.001 (ANOVA)
OCDS-G Score mean (sd)	1.39 (1.43)	18.9 (7.18)	<.001 (ANOVA)
BDI mean (sd)	3.23 (4.13)	14.17 (8.67)	<.001 (ANOVA)
Barratt Impulsivity Scale total score mean(sd)	42.58 (3.93)	44.36 (3.37)	.06 (ANOVA)

*School education level: none = no formal school certificate, low = 9 to 10 years German basic school level “Hauptschule”, mid = 10 years German mid level “Realschule”, high = 12 to 13 years German high level “Gesamtschule” or “Gymnasium” **at start of hospitalization

### Clinical assessments

In order to verify inclusion and exclusion criteria, patients and controls were assessed using the structured interview for DSM-IV criteria, the Mini-International Neuropsychiatric Interview [23]. The substance dependency-related scales used in this study were the Alcohol Use Disorders Identification Test [24], the German version of the Obsessive Compulsive Drinking Scale [25], the Fagerström Scale for Nicotine dependency [26] and the European Addiction Severity Index [27]. In order to assess additional symptom dimensions, all subjects completed the self-rating scales set forth in the Beck Depression Inventory [28] and the Barratt Impulsivity Scale [11, 29].

For patients receiving clomethiazole (elimination half-life 3–5 h) at least 24 h had elapsed between the last administration and the EEG assessment. Patients treated with clonazepam were scheduled for the EEG assessment as soon as they reached an estimated plasma level of < .05 mg/l, in accordance with its elimination half-life of 40 h and 1 day additional wash-out time [30]. Two patients received zopiclone the night before the assessment. With a half-life of 5 h, and the assessments taking place at more than 2.5 half-life, we do not expect this to have affected our results [31]. EEG and clinical assessments were conducted at least 10 days after the start of alcohol detoxification.

Three months after the EEG assessment, patients were re-contacted in a telephone survey inquiring as to whether or not they continued to be abstinent. Interviews were conducted by a physician (CMR) known to the patients from their time at the hospital, and the occurrence of a relapse was determined based on the information given by the patients during the interview. A relapse was diagnosed when patients reported a return to alcohol consumption rates at or near the level of their pre-detoxification use. The telephone survey did not include additional physical or psychological investigations.

### EEG and event-related potential task

The EEG assessments were conducted with subjects seated in a reclining chair in an electrically shielded room. The assessment was split into four runs of about 13 min each. Subjects with nicotine dependency were asked if they craved nicotine, and allowed to smoke following the first two runs in order to minimize the effects of acute nicotine deprivation on the EEG signals.

**Fig. 1:**
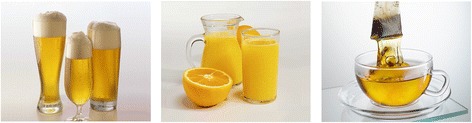
Visual stimuli showing the beer, the juice and the tea stimuli

Subjects were presented with three stimuli which depicted either three freshly drawn glasses of beer, an ensemble of a mug and a glass of orange juice or a cup of tea with the tea bag just being withdrawn. All three stimuli were licensed from a provider of professional food images (stockfood.de) for use as stimuli in research, and adjusted to match each other in hue and luminance. Stimuli were selected from among a variety of images via a pre-study in which 20 alcohol dependent patients rated the images based on popularity and craving potential. Among alcohol-related stimuli it turned out that beer scored the highest in popularity, followed by vodka and wine. Beer pictures which patients associated with brands from southern Germany scored low in popularity, and were therefore not taken into consideration. In selecting the neutral tea and juice stimuli it proved to be of relevance not to choose stimuli which patients may associate with alcoholic drinks. The selected pctures may be looked up and viewed by their reference numbers at stockfood.de; tea: 277227, orange juice: 00647384, beer: 00057014 (Fig. 1).

In each of the two experimental task conditions, 624 pictures were presented on a 14 in. computer monitor in pseudo-randomized order in two runs of 312 pictures each, for a duration of 500 ms per picture, with a 2500 ms inter-stimulus interval (+/- 500 ms). In the Go condition, subjects had to press a button with their right index finger upon appearance of the beer (*N* = 2 × 31, 12.5 %) and orange juice (*N* = 2 × 31, 12.5 %) stimuli, and had to ignore the frequent tea stimuli (*N* = 2 × 250, 75 %). In the NoGo condition, subjects had to press a button with their right index finger upon appearance of the frequent tea stimulus, and had to ignore the rare beer and orange juice stimuli. All stimuli were presented using Presentation software (Neurobehavioral Systems Inc.). In the Go condition, subjects were instructed to “please press the button whenever and only when you see the beer or the orange juice picture. Please ignore the tea picture”. In the NoGo condition, subjects were instructed to “please press a button whenever and only when you see the tea picture. Please ignore the beer and the juice pictures.” In subjects with a high amount of artifacts during data acquisition, additional runs were conducted in order to ensure that a sufficient amount of segments entered the single subject averages. This had to be done for six control subjects and three patients.

Each subject’s EEG was recorded using a 32 channel electrode cap with tin electrodes positioned according to the extended 10/20 system, with electrodes referenced to linked earlobes and two bipolar channels used to monitor horizontal and vertical eye movements. EEG data were amplified using a 32 channel Neuroscan Synamps amplifier in the frequency range of 0.03 Hz to 30 Hz at a sampling rate of 100 Hz. Electrode impedances were kept < 5 kΩ. EEG data analysis was performed using Neuroscan Edit (v4.2) and Brain Vision Analyzer (V1.05) software.

The EEG data were screened for sequences containing complex artifacts (i.e. clenching teeth or swallowing) which were then excluded from further analysis. Blink artifacts were corrected using the method described by Semlitsch [32], as implemented in Scan V.4.2 software. Data were segmented into intervals ranging between -100 ms and 1000 ms relative to stimulus onsets for segments with valid behavioral data. Segments were corrected for the -100 ms to 0 ms baseline interval, and segments exceeding +/- 50 μV shifts in EEG data were excluded from further analysis. Single subject averages were computed separately for stimulus categories and conditions.

Here we report data from electrodes O1 and O2. Single subject average curves were analyzed for event-related components P100 and N170 amplitudes. The mean number of segments in averages ranged between 322 (sd = 94.2, Go condition, patients) and 370 (sd = 84.7, tea, Go condition, controls) for the frequent tea stimulus, and 34 (sd = 8.5, beer, NoGo condition, patients) and 46 (sd = 11.1, juice, Go condition, controls) for the infrequent beer and juice stimuli.

### Data analysis

Behavioral data were analyzed with regard to error percent data for each stimulus under both task conditions using t-statistics. Amplitudes and latencies of P100 and N170 components at electrodes O1 and O2 were analyzed using repeated measurement MANOVAs with group (patients/controls) as between factor and electrode (O1, O2), stimulus (tea, orange juice, beer) and condition (Go [press for tea], NoGo [press for orange juice and beer]) as within factors. Statistical analyses were computed using statistical software SPSS V.22 (IBM Corp., New York). Analyses of ERPs comparing relapsing and non-relapsing patients were conducted separately for the two conditions as group (between factor: with/without relapse), by electrode (within factor: O1, O2) and by stimulus (within factor: tea, orange juice, beer) MANOVAs in order to reduce the complexity of analyses comparing groups with low numbers of subjects. MANOVA results are reported for within-subjects multivariate tests with Greenhouse-Geisser adjusted p-values. BDI scores were introduced as covariate in additional MANCOVA analyses in order to explore the influence of depression on ERP results.

## Results

### Subject characteristics

Details of subject characteristics are given in Table 1. Patients and controls did not differ with regard to age, gender, education or handedness. Mean breath alcohol concentration at hospitalization was 1.15 1 per mille (SD = 1.3), and mean years of alcohol dependency was 12 years (SD = 8). The mean number of previous inpatient detoxification treatments among patients was 4.9. On questionnaires and in interviews, patients scored higher than controls in terms of smoking, alcohol use, compulsive drinking and depression symptoms, but not in in terms of impulsivity as measured by the Barratt Impulsivity Scale. Relapse assessment was successfully achieved in 23 out of 30 patients. Eleven patients remained abstinent from alcohol and 12 patients relapsed within the 3-month evaluation period.

### Behavioral results

With regard to the Go and NoGo conditions, the amount of errors was slightly larger for patients under both conditions. Differences between patients and controls, however, failed to reach the level of statistical significance under both conditions and all three stimuli. A table on performance data is given in Table 2.

**Table 2:** Behavioral data

Condition	Stimulus	Patient m (sd)	Control m (sd)
Go (% errors)	Tea (non-target)	9.42 (9.93)	6.78 (6.19)
	Beer (target)	5.32 (6.30)	4.71 (6.97)
	Juice (target)	10.70 (8.10)	8.93 (6.18)
NoGo (% errors)	Tea (target)	6.59 (7.05)	7.09 (5.91)
	Beer (non-target)	24.79 (15.08)	22.46 (13.06)
	Juice (non-target)	17.04 (12.03)	13.38 (10.20)

### ERP results

**Fig. 2:**
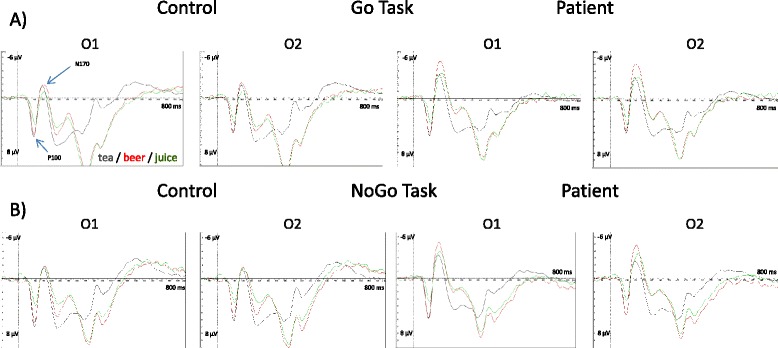
Grand averages of event-related responses in patients and controls to tea, orange juice and beer stimuli (**a**) in the Go and (**b**) the NoGo condition at occipital electrodes O1 and O2

**Fig. 3:**
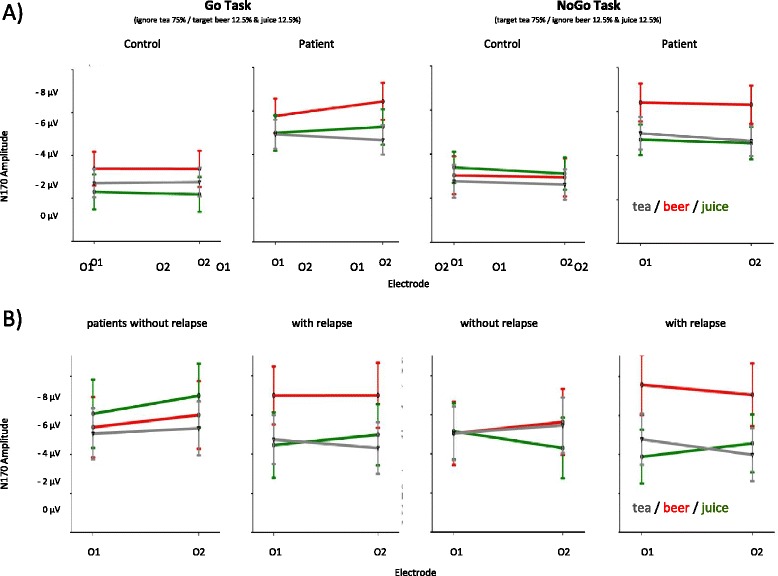
Mean and standard errors of the N170 component amplitudes of event-related responses to tea, orange juice and beer stimuli in the Go and NoGo condition at occipital electrodes O1 and O2 for patients and controls (**a**) and for patients with and without 3-months relapse (**b**)

ERP grand average curves for patients and controls are given in Fig. 2. Figure 3 shows amplitudes of the N170 component in patients and controls, and for relapsing and non-relapsing patients. The means and standard deviations of the occipital P100 and N170 component amplitudes and latencies are reported in Table 3. Details on analysis of variance and covariance results may be found in the Additional file 1.

**Table 3:** P100 and N170 component amplitudes and latencies of patients and controls

		Amplitude (mean/sd, μV)				Latency (mean/sd, ms)			
		Control		Patient		Control		Patient	
		O1	O2	O1	O2	O1	O2	O1	O2
p100	go beer	6.52 (4.42)	6.01 (4.35)	6.24 (3.70)	5.35 (3.70)	96.67 (13.42)	97.87 (18.90)	95.33 (11.96)	95.93 (11.55)
	go juice	4.82 (3.84)	4.58 (3.82)	4.35 (3.40)	4.19 (3.50)	100.47 (19.59)	99.87 (20.05)	100.73 (18.43)	95.87 (16.34)
	go tea	7.06 (4.49)	6.14 (4.12)	6.09 (3.64)	5.71 (3.50)	95.60 (19.44)	98.93 (11.93)	96.47 (10.34)	96.27 (10.76)
	no go beer	7.24 (4.77)	6.77 (4.32)	7.02 (3.78)	6.44 (3.57)	99.27 (14.14)	98.60 (11.99)	96.60 (12.14)	97.20 (13.44)
	no go juice	5.39 (3.32)	5.23 (3.15)	4.37 (3.40)	4.42 (3.12)	97.47 (23.00)	100.07 (17.62)	102.00 (19.87)	101.73 (18.91)
	no go tea	8.24 (5.60)	7.12 (5.06)	6.80 (3.84)	6.34 (3.96)	98.07 (14.57)	97.13 (11.72)	98.80 (10.75)	97.40 (15.82)
n170	go beer	−3.38 (3.60)	−3.37 (3.44)	−5.78 (4.92)	−6.44 (5.54)	153.80 (20.56)	148.73 (19.27)	152.27 (33.61)	147.53 (18.71)
	go juice	−2.18 (3.64)	−2.30 (3.38)	−5.27 (5.11)	−4.99 (5.29)	147.60 (27.78)	149.73 (32.51)	148.40 (28.50)	144.13 (31.09)
	go tea	−2.82 (3.20)	−2.76 (3.17)	−4.93 (4.04)	−4.67 (4.14)	145.20 (18.75)	149.87 (13.74)	147.53 (17.48)	147.13 (17.54)
	no go beer	−3.03 (3.74)	−2.94 (3.73)	−6.41 (5.47)	−6.31 (5.54)	148.07 (19.01)	146.20 (17.54)	149.40 (20.50)	150.00 (20.91)
	no go juice	−3.39 (3.17)	−3.11 (3.03)	−4.72 (4.44)	−4.56 (4.83)	148.53 (24.77)	146.53 (21.85)	155.07 (30.37)	155.53 (30.22)
	no go tea	−2.77 (3.82)	−2.61 (2.95)	−5.01 (4.35)	−4.66 (4.45)	148.93 (14.99)	146.93 (22.11)	150.53 (16.71)	146.67 (24.63)

### Patient vs. control

#### P100 and N170 latencies

An analysis of latencies in two stimulus type (within factor: Tea, Juice, Beer) x condition (within factor: Go, NoGo) x electrode (within factor: O1,O2) x group (between factor: patients, controls) MANOVAs for the P100 and the N170 components yielded no significant results.

#### P100 amplitudes

With regard to P100 amplitudes, we found a condition (Go/NoGo) main effect (*F*_(1,58)_ = 7.8, *p* = .007), a main effect for stimulus type (tea, juice,beer: *F*_(1.8,104.9)_ = 41.1, *p* < .001) and a stimulus x electrode interaction (*F*_(1.9,109.4)_ = 6.4, *p* = .003). P100 amplitudes were larger in the NoGo as compared to the Go condition. The juice stimulus showed lower amplitudes as compared to the tea and beer stimuli, regardless of condition and group. O1 electrode amplitudes were larger than O2 amplitudes for tea and beer but not for the juice stimuli. For P100 amplitudes we found no results involving the group factor.

#### N170 amplitudes

With N170 amplitudes we found a main effect for group (patients, controls: *F*_(1,58)_ = 7.3, *p* = .009), a main effect for stimuli (tea, juice, beer, *F*_(1.9,113.9)_ = 6.3, *p* = .003) and a three-way condition (go/nogo) x stimulus type (tea, juice, beer) x group (patients, control) interaction (*F*_(1.8,106.5)_ = 4.0, *p* = .024). N170 amplitudes were larger (i.e. more negative) in patients as compared to controls. The beer stimulus resulted in more negative amplitudes as compared to the tea and juice stimuli. The three-way interaction of within factor condition (Go/NoGo) x within factor stimulus type (tea, juice, beer) x between factor group (patient, control) was attributable to larger beer stimulus amplitudes in patients as compared to controls—and more so in the NoGo as compared to the Go condition—indicating larger (i.e. more negative) beer stimulus amplitudes when patients had to react to the tea and ignore the beer and juice stimuli. Significant effects related to group were further examined under separate analyses for each task condition with two within factor stimulus type (tea, juice, beer) x between factor group (patient, control) MANOVAs for the Go and the NoGo condition. The main effect of group with heightened amplitudes in patients remained statistically significant in both conditions (Go *F*_(1,59)_ = 7.4, *p* = .008; NoGo *F*_(1,58)_ = 6.0, *p* = .017). Upon closer examination of the three-way interaction involving the group factor with these two MANOVA analyses conducted separately for the Go and the NoGo condition, the stimulus type x group interaction in the Go condition for the N170 component was not significant (*F*_(1.8,108.9)_ = 0.79, *p* = .445) whereas the interaction was still significant in the NoGo condition (*F*_(1.9,112.5)_ = 4.2, *p* = .018). Therefore, the effect of heightened N170 amplitudes in response to the beer stimulus in the patients as compared to controls was attributable to the NoGo condition, where subjects had to react to the tea and ignore the juice and beer stimuli.

#### Depression as a covariate

Since depression symptoms may act as a confounding variable, we conducted additional analyses of variance, replicating previous analyses which included Beck Depression Inventory scores as covariate. For the P100 amplitudes in the between factor group (patient/control) x within factor condition (Go/NoGo) x within factor stimulus type (tea/juice/beer) x within factor electrode (O1/O2) MANCOVA with BDI scores as covariate, we replicated the main effects of stimulus and condition but not the stimulus x electrode interaction. No other effects were statistically significant. With P100 latencies we found a stimulus x electrode x BDI score interaction (*F*_1,71; 97.29_ = 3.70, *p* = .035). While P100 latencies may vary with different stimuli at the two occipital electrodes with depression scores, this effect is unrelated to alcohol addiction in our study.

With N170 amplitudes and BDI scores as covariate in the MANCOVA analysis, we replicated the main effect for group. The main effect for stimulus type, however, was no longer significant. The three-way interaction of group x stimulus type x condition was still significant (*F*_1.84; 104.75_ = 3.63, *p* = .034). No other effects were statistically significant. Our follow-up analysis of the the significant three-way interaction effect in N170 latencies in the Go and the NoGo conditions separately in group x electrode x stimulus MANCOVA analyses with BDI as covariate, replicated the main effect for group only in the Go condition. No effects involving the BDI scores were found. The stimulus type x group interaction in the NoGo condition was not replicated in the BDI covariance analysis. With N170 latencies we found no effects in the additional BDI covariance analyses.

#### Relapse assessment

Patients with (*n* = 12) and without (*n* = 11) 3-month relapse did not differ with regard to age, gender, education or preferred form of alcohol consumption. Additionally, they did not differ in clinical scales (Fagerström, AUDIT, OCDS-G, ASI, MINI, BIS), apart from the Beck Depression Inventory (BDI). When measured at the time of the EEG assessment, patients who stayed abstinent over the next 3 months scored 9.0 (sd = 7.4) BDI points versus 18.3 (sd = 6.5) points in patients who relapsed. Details on subject characteristics and ERPs in relapsing and non-relapsing patients are given in Table 4.

**Table 4:** Subject characteristics: patients with and without relapse

	Without relapse (*n* = 11)	With relapse (*n* = 12)	p =
Age y(std)	48.27 (8.67)	42.17 (7.60)	.086 (ANOVA)
Handedness (n, right)	11/11	12/12	n.a.
Gender (n: f/m)	2/9	9/9	.545 (Chi^2^)
Nicotine dependency (n)	7	11	.131 (Chi^2^)
Fagerström Score mean (sd)	5.00 (4.17)	6.67 (3.37)	.302(ANOVA)
School (n:without/low/mid/high*):	0,5,2,4	1,5,2,4	.811 (Chi^2^)
Preference drink (n:beer/wine/other)	6/3/2	10/1/1	.318 (Chi^2^)
Health-related problems (n)	14 (15.36)	9 (12.95)	.445 (ANOVA)
Breath alcohol concentration in per mille (sd)**	0.47 (0.60)	1.36 (1.34)	.055 (ANOVA)
AUDIT mean (sd)	20.82 (6.11)	24.92 (5.74)	.174 (ANOVA)
OCDS-G Score mean (sd)	16.45 (8.65)	20.33 (5.74)	.215 (ANOVA)
BDI mean (sd)	9.00 (7.43)	18.25 (6.45)	.004* (ANOVA)
Barratt Impulsivity Scale total score mean (sd)	66.27 (4.92)	66.58 (3.80)	.866 (ANOVA)
Previous detoxifications, mean (sd)	1.55 (1.51)	7.17 (8.91)	.052 (ANOVA)

*School education level: none = no formal school certificate, low = 9 to 10 years German basic school level “Hauptschule”, mid = 10 years German mid level “Realschule”, high = 12 to 13 years German high level “Gesamtschule” or “Gymnasium” **at start of hospitalization

Figure 3 shows ERP grand average curves of relapsing and non-relapsing patients with regard to the occipital P100 and N170 components. The P100 and N170 ERP component amplitudes and latencies were submitted to separate MANOVAs with condition (Go/NoGo) and electrode (O1/O2) as within factors and group (relapse/non relapse) as between factor in the 23 patients which were successfully interviewed 3 months following the initial assessments. No significant effects emerged for the analysis of P100 and N170 latencies.

In the between factor group (relapse/abstinence) x within factor stimulus (tea, juice beer) x within factor electrode (O1/O2) MANOVAs of P100 amplitudes in the Go and the NoGo condition separately, we found a main effect for stimuli but no effect related to relapse status. Juice stimuli were associated with larger amplitudes in the Go (*F*_(1.9,39.9)_ = 11.2, *p* < .001) and the NoGo condition (*F*_(1.7,36.0)_ = 12.3, *p* < .001). In the analysis of N170 amplitudes in a between group (relapse/abstinence) x within stimulus (tea, juice, beer) x within electrode (O1/O2) MANOVAs separately for the Go and the NoGo condition we found a main effect for stimuli only in the NoGo condition (*F*_(1.8,39.1)_ = 7.4, *p* = .002). This effect was attributable to heightened beer stimulus amplitudes. Additionally, we found a stimulus x group interaction in the NoGo condition with heightened amplitudes in response to the beer stimulus in patients who relapsed during the 3-month evaluation period (*F*_(1.9,39.2)_ =4.6 *p* = .018). Details of amplitudes and latencies for relapsing and non-relapsing patients are given in Table 5.

**Table 5:** P100 and N170 component amplitudes and latencies of patients with and without relapse *n* = 23

		Amplitude (mean/sd, μV)				Latency (mean/sd, ms)			
		Without relapse		With relapse		Without relapse		With relapse	
		O1	O2	O1	O2	O1	O2	O1	O2
p100	go beer	5.53 (3.25)	4.40 (2.94)	7.96 (4.10)	7.10 (4.52)	96.18 (8.60)	95.64 (8.98)	101.00 (8.76)	100.67 (10.93)
	go juice	3.63 (3.00)	3.45 (2.78)	5.78 (3.97)	5.63 (4.49)	110.36 (14.47)	97.45 (18.40)	100.50 (17.38)	96.00 (16.45)
	go tea	5.36 (4.16)	5.07 (3.87)	7.27 (3.97)	6.89 (3.89)	92.91 (12.28)	94.00 (13.11)	100.00 (8.78)	110.36 (14.47)
	no go beer	7.12 (3.33)	5.98 (3.32)	7.84 (4.81)	7.53(4.46)	94.36 (11.86)	96.54 (17.98)	101.33 (11.45)	100.33 (11.63)
	no go juice	4.30 (3.93)	3.77 (3.08)	5.25 (3.10)	5.58 (3.58)	99.09 (22.63)	98.18 (20.03)	106.17 (14.86)	102.67 (20.70)
	no go tea	6.01 (4.20)	5.19 (4.14)	8.16 (4.15)	7.68 (4.61)	98.18 (12.63)	94.91 (10.86)	101.67 (11.24)	100.00 (22.53)
n170	go beer	−5.39 (3.66)	−6.02 (4.73)	−7.00 (6.18)	−7.01 (6.54)	169.45 (41.78)	146.55 (14.56)	149.83 (13.66)	150.83 (10.63)
	go juice	−6.08 (6.01)	−7.01 (5.65)	−4.46 (5.62)	−5.00 (5.23)	164.55 (23.22)	158.18 (29.78)	144.00 (22.95)	136.67 (26.00)
	go tea	−5.07 (3.85)	−5.34 (3.47)	−4.75 (4.75)	−4,31 (5.40)	146.91 (23.07)	148.55 (23.27)	146.50 (10.17)	146.33 (9.79)
	no go beer	−5.05 (5.20)	−5.63 (4.87)	−7.58 (5.52)	−7.07 (6.18)	147.45 (26.02)	148.18 (24.21)	152.67 (26.02)	153.00 (12.25)
	no go juice	−5.15 (4.31)	−4.30 (4.07)	−3.88 (5.17)	−4.57 (6.00)	145.64 (31.58)	152.91 (33.98)	163.17 (29.82)	157.17 (30.04)
	no go tea	−5.05 (4.41)	−5.47 (3.83)	−4.78 (4.70)	−3.98 (5.39)	150.91 (17.60)	148.73 (24.09)	148.83 (13.28)	140.17 (26.08)

#### Depression as a covariate in relapse assessment

Since depression symptoms may act as a confounding variable, we conducted additional MANCOVA analyses, replicating previous analyses which included Beck Depression Inventory scores as covariate. For the P100 amplitudes in the between factor group (with/without relapse) x within factor stimulus type (tea/juice/beer) x within factor electrode (O1/O2) MANCOVAs with BDI scores as covariate in the Go and the NoGo condition separately, we found main effects for group in both conditions. Additionally, we found main effects for the BDI scores in both analyses. Heightened BDI scores tended to be associated with shorter P100 latencies in the Go as well as in the NoGo condition. Introduction of the BDI covariate revealed a relapse group main effect. Relapsing patients showed longer P100 latencies when BDI acted as covariate (P100 Go condition: depression main effect *F*_[1, 20]_ = 10.48, *p* = .004; P100 NoGo condition: depression main effect *F*_[1, 20]_ = 7.44, *p* = .013).

## Discussion

The main finding of the present study is that occipital N170 amplitudes in response to the addiction-related beer stimulus were elevated in recently detoxified patients with alcohol dependence when the stimulus had to be ignored and attention was focused on the non-addiction-related tea stimulus. The specificity of this result is further supported by the fact that the increase in amplitude did not occur to the same extent under the other to-be-ignored NoGo stimulus, i.e. the juice picture in our experiment. To our knowledge, this effect has not been reported previously. The relevance of this finding for relapses in patients is supported by our second major finding. Patients who relapsed in the 3 months following detoxification had larger occipital NoGo N170 amplitudes in response to the alcohol- related stimulus as compared to those who remained abstinent at the time of the EEG assessment.

This latter result is in line with recently published evidence by Petit et al. [12], who reported lowered ERP amplitudes to be related to successful 3-month abstinence among recently detoxified alcohol addicts. Although Petit et al. based their results on parameters which substantially differ from those applied in our study, both studies imply that a lower reactivity to addiction-related stimuli is correlated with abstinence success.

EEG studies exploring cue reactivity have mainly focused on ERP components with peaks at frontal to parietal sites, such as the P300 component. P300 amplitudes show larger amplitudes in response to addiction-related cues [10, 33], but have only rarely been investigated at occipital electrodes or related to visual processing of the P100 component [34]. It is of interest to note that the study by Bloom et al. [33] reported increased amplitudes only to target stimuli which elicit the P300 component, but not to non-target stimuli in their experiment. When considering the relevance of electro-cortical responses to NoGo stimuli, these are usually investigated in tasks targeting frontal control processes, for example in experiments on the NoGo-anteriorisation effect studied by the Fallgatter group (i.e. [35]).

Maurage et al. [13, 14] studied stimulus processing in alcohol dependent subjects by analyzing the occipital N170 component in response to emotional face stimuli, and found increased latencies and reduced amplitudes in patients. Here, we found prolonged P100 latencies only in the relapse assessment and when the effect of depression was controlled for in the covariance analyses. While this effect was not seen in the covariance analyses comparing patients and controls, it may be attributable to the group of the more severely affected patients who relapsed within 3 month following detoxification. Condition and stimulus-related P100 amplitude main effects may be attributable to physical aspects of the stimuli, which are unrelated to the group factor. With regard to the N170 component, our study, however, does not replicate latency differences, and amplitudes are not diminished in the processing of visual stimuli in our patients, but rather heightened in the N170 component amplitudes, and significantly so, with the to-be-ignored NoGo beer stimulus. This may indicate a specific sensitivity of occipital cortical reactions to NoGo stimuli which previous research focusing on frontal control processes associated with response inhibition to rare stimuli may have overlooked.

The experimental design of our study controlled for the condition relevance and addiction relevance of the stimuli by requiring subjects to press a button either upon the occurrence of the rare beer and juice stimuli in the Go condition (hence controlling for addiction relevance), or upon the occurrence of the frequent tea stimulus in the NoGo condition (hence controlling for target action). While in neuroscience research NoGo conditions constitute conditions requiring executive control in the inhibition of actions, theories on inhibition processes concentrate on networks involving sub-cortical areas interacting with frontal brain structures [36]. A recent study by Caharel et al. found that amplitude modulations of the N170 component in a Go-NoGo task were related to behavior only from 200 ms onward. The N170 amplitude was found to be modulated in specific task conditions, but this effect was not related to behavior in this experiment [37]. Although Maurage et al. recently demonstrated that executive attention control processes are of specific relevance to alcohol addicted patients [38], we suggest that the observed amplitude increase in patients’ N170 component in response to the beer stimulus under the NoGo condition is associated with incentive salience contributions to the visual processing of alcohol-related cues in this condition. It may be that the present study sheds no light on the exact nature of the interaction between the addiction specific significance of the beer stimulus and the attention control processes inherent in the NoGo condition; but our findings may constitute an argument in favor of more specifically focusing on the processing of drug-related stimuli which have to be ignored.

The main effect for group indicating heightened N170 ERP component amplitudes in patients regardless of condition type may be related to the timing of the initial assessment, which took place closely after the patients’ detoxification from alcohol. In a rat model of withdrawal, Cheaha et al. [39] recently reported increased gamma band power following ethanol withdrawal in rats, and interpreted these in support of a serotonergic hypofunction. In alcohol dependent patients, relapse was associated with increased high frequency beta band EEG [40]. A study by Polo et al. found increased frontal P3a ERP amplitudes in response to NoGo stimuli among patients abstaining from alcohol [41]. While some EEG and ERP abnormalities have been considered as endophenotypes in alcoholism [8], in our study only the N170 amplitudes in response to the NoGo beer stimulus were related to addiction and to the 3-month relapse outcome. Therefore, we suggest that the heightened NoGo N170 amplitudes in response to the beer stimulus are specifically related to a high risk of relapse, while the NoGo N170 amplitude main effect may be due either to heightened post-withdrawal cortical excitability or to a general feature of altered cortical reactivity in subjects at risk for alcohol dependency [42].

While recent neuroimaging research has focused on frontal and sub-cortical processing in addiction research [43, 44], our results may be viewed as partly compatible with recent data on cue reactivity in cocaine addicted subjects reporting occipital BOLD activation in response to cocaine cues, as related to relapses in patients [45]. Conversely, in the same group, those patients with high treatment motivation exhibited lower cocaine cue reactivity in the occipital cortex regions [46]. Although another study [47] found no connection between drug-related occipital BOLD cue reactivity and transition to heavy drinking, we suggest that this may be because the predictive effect of drug-related cues may be confined to relapse propensity and cues in non-target contexts. The relevance of non-target signals for relapse is further supported by recent animal research demonstrating increased relapse-related behavior in rats when exposed to alcohol-related context stimuli [48]. While the translational bridge from conditioning experiments in rats to our study in patients is large, this finding does suggest that non-target stimulus contexts are relevant in understanding relapse-related behavior in alcohol dependent patients.

Socio-demographic control variables like age, gender and years of education did not differ between patients and controls. The control group was recruited to match the patients in these variables and thus, by design, the controls in our study were not primarily recruited from university campuses. The amount of health-related problems and the preferred kind of drinks did not differ either, although there is some evidence to suggest that these variables may differ in larger samples [49]. Clinical symptoms, on the other hand, were found to be associated with addiction-related measures (AUDIT, ODCS) and nicotine use (Fagerström). With regard to psychological symptom dimensions, impulsivity scores did not differ significantly between patients and controls; and did not differ between relapsing and non-relapsing patients. Patients did, however, exhibit heightened BDI scores as compared to controls; and the BDI scores of relapsing patients were higher than those of non-relapsing patients. Indeed, the BDI score differential between relapsing and non-relapsing patients was substantial, highly significant and reached the range of clinical relevance [50]. This result agrees with a broad range of evidence linking depression to relapses in the treatment of alcoholism [51, 52].

In additional analyses of covariance which included BDI scores, we aimed to assess whether heightened depression scores may explain our main finding of elevated N170 amplitudes under the NoGo condition, or whether the scores otherwise interact with the ERP data. Our results indicate that depression scores only interacted with early P100 ERP results in an electrode x stimulus x depression interaction, which is unrelated to the group factor. As regards the N170 component, depression did not interact with ERP results, and the main result of the group x condition x stimuli interaction was still significant in the omnibus analysis. The lack of significant interactions in the follow-up analyses comparing patients and controls under both task conditions may be attributed to the increased complexity of these MANCOVA analyses.

While depression did not show a significant association with ERP amplitude data in any of the analyses, this may be because depression is not associated with the specific N170 ERP cue reactivity result of our study. Depression may interact with early occipital P100 latencies in patients, but overall it seems to constitute an independent factor contributing to relapse behavior following alcohol detoxification. A lager study sample size may be needed to further disentangle and differentiate between the respective contributions made by depression and cue reactivity in occipital ERPs to relapse behavior in detoxified alcohol patients.

Two methodological considerations should be kept in mind when interpreting our results. The chosen sampling rate of 100 Hz may be considered as sufficient to assess the intended amplitude peaks of the P100 and the N170 ERP components. This may, however, prohibit the detection of small changes in latencies of ERP component peaks. Researchers conducting a replication of the current study may therefore wish to use a higher sampling rate. Another methodological consideration is our focus on one stimulus per category. Specific picture features may have influenced the early occipital P100 component which we found between stimuli. These, however, emerged regardless of group (patient/control) and relapse status. Care was taken to select stimuli in a pre-study assessment among patients with alcohol dependence so as to eliminate associations between neutral juice stimuli and alcoholic drinks; but it is possible that some stimulus-specific parameters with effects on the P100 have remained.

## Conclusions

In conclusion, the results of our study indicate the relevance of addiction-related stimuli in situations that do not require directed attention for the automated processing of alcohol-related cues. Action- related control is focused on the frequent target tea stimulus in the NoGo condition; and while the alcohol-related stimulus in addicted patients receives increased cortical processing this may build a gap for the addiction-related stimulus to be integrated into further cortical processing. This probably induces craving which may then lead to behavioral changes conducive to relapse-related actions which the patient is unprepared to voluntarily inhibit. ERPs may reflect the propensity for addicted patients to relapse within the first 3 months following alcohol detoxification. Finally, our data underscore the relevance of heightened depression symptoms for relapse risk in patients with alcohol dependence.

## Additional file

Additional file 1: P100 and N170 component MANCOVA analyses with BDI scores as covariate in patients and controls and in the relapse assessments. (DOCX 36 kb)
