# Supplementary material for: Occipital event-related potentials to addiction-related stimuli in detoxified patients with alcohol dependence, and their association with three-month relapse
Source: BMC Psychiatry. 2016 Mar 21;16:74. doi: 10.1186/s12888-016-0782-0 (PMC4802663; doi:10.1186/s12888-016-0782-0)
Supplement: Additional file 1: — P100 and N170 component MANCOVA analyses with BDI scores as covariate in patients and controls and in the relapse assessments. (DOCX 36 kb) [file 12888_2016_782_MOESM1_ESM.docx]

**Patient / control: amplitude data**

| **P100** |  | F | Df | Sig. |
| --- | --- | --- | --- | --- |
| Main effects | group | 1.26 | 1/57 | .266 |
| n =30/30 | depression | 0.67 | 1/57 | .417 |
|  | electrode | 1.40 | 1/57 | .241 |
|  | stimulus | 12.69 | 1.81/103.24 | **.000**** |
|  | condition | 6.97 | 1/57 | **.011*** |
| Interactions | group * electrode | 0.06 | 1/57 | .806 |
|  | group * stimulus | 1.03 | 1.81/103.24 | .355 |
|  | group * condition | 0.13 | 1/57 | .721 |
|  | electrode * stimulus | 2.21 | 1.96/111.59 | .115 |
|  | electrode * condition | 0.39 | 1/57 | .533 |
|  | electrode * depression | 0.00 | 1/57 | .985 |
|  | stimulus * condition | 0.39 | 1.81/103.32 | .656 |
|  | stimulus * depression | 0.40 | 1.81/103.24 | .654 |
|  | condition * depression | 1.36 | 1/57 | .249 |
|  | group * electrode * stimulus | 1.38 | 1.96/111.59 | .256 |
|  | group * electrode * condition | 0.38 | 1/57 | .542 |
|  | group * stimulus * condition | 1.91 | 1.81/103.32 | .157 |
|  | electrode * stimulus * condition | 0.91 | 1.65/93.95 | .390 |
|  | electrode * stimulus * depression | 2.18 | 1.96/111.59 | .119 |
|  | electrode * condition * depression | 0.33 | 1/57 | .565 |
|  | stimulus * condition * depression | 2.62 | 1.81/103.32 | .083 |
|  | condition * stimulus * electrode * depression | 0.87 | 1.65/93.95 | .403 |
|  | condition * stimulus * electrode * group | 0.952 | 1.65/93.95 | .375 |

| **N170** |  | F | Df | Sig. |
| --- | --- | --- | --- | --- |
| Main effects | group | 4.30 | 1/57 | **.043*** |
| n =30/30 | depression | 0.00 | 1/57 | .991 |
|  | electrode | 0.00 | 1/57 | .954 |
|  | stimulus | 0.19 | 1.94/110.66 | .821 |
|  | condition | 0.28 | 1/57 | .598 |
| Interactions | group * electrode | 0.08 | 1/57 | .781 |
|  | group * stimulus | 0.11 | 1.94/110.66 | .892 |
|  | group * condition | 0.01 | 1/57 | .918 |
|  | electrode * stimulus | 2.21 | 1.96/111.59 | .115 |
|  | electrode * condition | 2.25 | 1/57 | .351 |
|  | electrode * depression | 0.08 | 1/57 | .781 |
|  | stimulus * condition | 0.04 | 1.84/104.75 | .950 |
|  | stimulus * depression | 3.09 | 1.94/110.66 | .051 |
|  | condition * depression | 0.23 | 1/57 | .636 |
|  | group * electrode * stimulus | 1.38 | 1.96/111.59 | .256 |
|  | group * electrode * condition | 1.10 | 1/57 | .299 |
|  | group * stimulus * condition | 3.63 | 1.84/104.75 | **.034*** |
|  | electrode * stimulus * condition | 0.91 | 1.65/93.95 | .390 |
|  | electrode * stimulus * depression | 2.18 | 1.96/111.59 | .119 |
|  | electrode * condition * depression | 0.88 | 1/57 | .351 |
|  | stimulus * condition * depression | 0.36 | 1.84/104.75 | .681 |
|  | condition * stimulus * electrode * depression | 0.87 | 1.65/93.95 | .403 |
|  | condition * stimulus * electrode * group | 0.95 | 1.65/93.95 | .375 |

**Patient / control : latency data**

| **P100** |  | F | Df | Sig. |
| --- | --- | --- | --- | --- |
| Main effect | group | 0.21 | 1/57 | .653 |
| n =30/30 | depression | 0.89 | 1/57 | .349 |
|  | electrode | 0.08 | 1/57 | .784 |
|  | stimulus | 3.02 | 1.83/104.50 | .058 |
|  | condition | 3.67 | 1/57 | .060 |
| Interactions | group * electrode | 0.82 | 1/57 | .370 |
|  | group * stimulus | 1.04 | 1.83/104.50 | .352 |
|  | group * condition | 2.92 | 1/57 | .093 |
|  | electrode * stimulus | 3.28 | 1.71/97.29 | .050 |
|  | electrode * condition | 0.16 | 1/57 | .689 |
|  | electrode * depression | 0.09 | 1/57 | .760 |
|  | stimulus * condition | 0.31 | 1.38/78.57 | .650 |
|  | stimulus * depression | 1.18 | 1.83/104.50 | .310 |
|  | condition * depression | 2.38 | 1/57 | .128 |
|  | group * electrode * stimulus | 2.90 | 1.71/97.29 | .070 |
|  | group * electrode * condition | 0.07 | 1/57 | .793 |
|  | group * stimulus * condition | 1.09 | 1.38/78.57 | .321 |
|  | electrode * stimulus * condition | 0.24 | 1.73/98.77 | .758 |
|  | **electrode * stimulus * depression** | **3.70** | **1.71/97.29** | **.035*** |
|  | electrode * condition * depression | 0.31 | 1/57 | .583 |
|  | stimulus * condition * depression | 0.57 | 1.38/78.57 | .504 |
|  | condition * stimulus * electrode * depression | 0.15 | 1.73/98.77 | .836 |
|  | condition * stimulus * electrode * group | 0.10 | 1.73/98.77 | .884 |

| **N170** |  | F | Df | Sig. |
| --- | --- | --- | --- | --- |
| Main effect | group | 0.36 | 1/57 | .552 |
| n =30/30 | depression | 0.29 | 1/57 | .593 |
|  | electrode | 0.34 | 1/57 | .565 |
|  | stimulus | 0.06 | 1.97/112.11 | .940 |
|  | condition | 0.95 | 1/57 | .333 |
| Interactions | group * electrode | 0.13 | 1/57 | .715 |
|  | group * stimulus | 0.23 | 1.97/112.11 | .789 |
|  | group * condition | 2.68 | 1/57 | .107 |
|  | electrode * stimulus | 0.68 | 1.65/94.21 | .483 |
|  | electrode * condition | 0.01 | 1/57 | .940 |
|  | electrode * depression | 0.00 | 1/57 | .981 |
|  | stimulus * condition | 1.94 | 1.37/78.26 | .163 |
|  | stimulus * depression | 0.33 | 1.97/112.11 | .717 |
|  | condition * depression | 0.72 | 1/57 | .399 |
|  | group * electrode * stimulus |  |  |  |
|  | group * electrode * condition | 0.52 | 1/57 | .472 |
|  | group * stimulus * condition | 1.23 | 1.37/78.26 | .285 |
|  | electrode * stimulus * condition | 1.08 | 1.93/110.12 | .343 |
|  | electrode * stimulus * depression | 0.58 | 1.65/94.21 | .530 |
|  | electrode * condition * depression | 0.00 | 1/57 | .966 |
|  | stimulus * condition * depression | 0.84 | 1.37/78.26 | .396 |
|  | condition * stimulus * electrode * depression | 0.25 | 1.93/110.12 | .769 |
|  | condition * stimulus * electrode * group | 0.08 | 1.93/110.12 | .917 |

**Patients with / without relapse: P100 latency data**

| **P100 go condition** |  | F | Df | Sig. |
| --- | --- | --- | --- | --- |
| Main effect | **group** | **5.84** | **1/20** | **.025*** |
| n =11/12 | **depression** | **10.48** | **1/20** | **.004**** |
|  | electrode | 0.09 | 1/20 | .756 |
|  | stimulus | 1.74 | 1.84/36.87 | .190 |
| Interactions | group * electrode | 0.84 | 1/20 | .368 |
|  | group * stimulus | 1.02 | 1.84 | .363 |
|  | electrode * stimulus | 2.15 | 1.38/36.87 | .155 |
|  | electrode * depression | 0.24 | 1/20 | .629 |
|  | stimulus * depression | 0.88 | 1.84/36.87 | .416 |
|  | group * electrode * stimulus | 0.27 | 1.38/22.75 | .633 |
|  | electrode * stimulus * depression | 1.13 | 1.38/22.75 | .530 |

| **P100 nogo condition** |  | F | Df | Sig. |
| --- | --- | --- | --- | --- |
| Main effect | **group** | **5.61** | **1/20** | **.028*** |
| n =11/12 | **depression** | **7.44** | **1/20** | **.013*** |
|  | electrode | 0.10 | 1/20 | .751 |
|  | stimulus | 1.37 | 1.94/38.93 | .265 |
| Interactions | group * electrode | 0.43 | 1/20 | .517 |
|  | group * stimulus | 0.01 | 1.94/38.93 | .989 |
|  | electrode * stimulus | 0.72 | 1.67/33.42 | .471 |
|  | electrode * depression | 1.18 | 1/20 | .289 |
|  | stimulus * depression | 0.35 | 1.94/38.93 | .698 |
|  | group * electrode * stimulus | 0.20 | 1.67/33.42 | .774 |
|  | electrode * stimulus * depression | 0.45 | 1.67/33.42 | .604 |

**Patients with / without relapse: N170 latency data**

| **N170 go condition** |  | F | Df | Sig. |
| --- | --- | --- | --- | --- |
| Main effect | group | 1.29 | 1/20 | .268 |
| n =11/12 | depression | 0.27 | 1/20 | .605 |
|  | electrode | 1.24 | 1/20 | .277 |
|  | stimulus | 0.41 | 1.62/32.54 | .624 |
| Interactions | group * electrode | 0.18 | 1/20 | .894 |
|  | group * stimulus | 1.24 | 1.62/32.54 | .295 |
|  | electrode * stimulus | 0.42 | 1.40/28.01 | .588 |
|  | electrode * depression | 0.01 | 1/20 | .894 |
|  | stimulus * depression | 0.06 | 1.62/32.54 | .902 |
|  | group * electrode * stimulus | 1.87 | 1.54/28.01 | .180 |
|  | electrode * stimulus * depression | 0.12 | 1.40/28.01 | .809 |

| **N170 nogo condition** |  | F | Df | Sig. |
| --- | --- | --- | --- | --- |
| Main effect | group | 1.75 | 1/20 | .200 |
| n =11/12 | depression | 2.14 | 1/20 | .158 |
|  | electrode | 0.75 | 1/20 | .394 |
|  | stimulus | 3.09 | 1.80/36.08 | .062 |
| Interactions | group * electrode | 1.56 | 1/20 | 0.226 |
|  | group * stimulus | 2.51 | 1.80/36.08 | .100 |
|  | electrode * stimulus | 0.75 | 1.32/26.47 | .428 |
|  | electrode * depression | 0.58 | 1/20 | .453 |
|  | stimulus * depression | 2.49 | 1.80/36.08 | .102 |
|  | group * electrode * stimulus | 1.05 | 1.32/26.47 | .334 |
|  | electrode * stimulus * depression | 1.25 | 1.32/26.47 | .286 |

**Patients with / without relapse: P100 amplitude data**

| **P100 go condition** |  | F | Df | Sig. |
| --- | --- | --- | --- | --- |
| Main effect | group | 0.76 | 1/20 | .391 |
| n =11/12 | depression | 0.35 | 1/20 | .557 |
|  | electrode | 0.74 | 1/20 | .548 |
|  | stimulus | 1.25 | 1.89/37.96 | .295 |
| Interactions | group * electrode | 0.02 | 1/20 | .947 |
|  | group * stimulus | 1.20 | 1.89/37.96 | .715 |
|  | electrode * stimulus | 3.29 | 1.16/23.28 | .274 |
|  | electrode * depression | 0.00 | 1/20 | .997 |
|  | stimulus * depression | 0.128 | 1.89/37.96 | .871 |
|  | group * electrode * stimulus | 0.46 | 1.16/23.28 | .711 |
|  | electrode * stimulus * depression | 2.36 | 1.16/23.28 | .360 |

| **P100 nogo condition** |  | F | Df | Sig. |
| --- | --- | --- | --- | --- |
| Main effect | group | 0.74 | 1/20 | .397 |
| n =11/12 | depression | 0.00 | 1/20 | .988 |
|  | electrode | 0.35 | 1/20 | .561 |
|  | stimulus | 1.07 | 1.74/34.87 | 0.34 |
| Interactions | group * electrode | 2.65 | 1/20 | .442 |
|  | group * stimulus | 1.44 | 1.74/34.87 | .783 |
|  | electrode * stimulus | 0.30 | 1.71/34.34 | .726 |
|  | electrode * depression | 0.00 | 1/20 | .986 |
|  | stimulus * depression | 1.58 | 1.74/34.87 | .222 |
|  | group * electrode * stimulus | 0.01 | 1.71/34.34 | .981 |
|  | electrode * stimulus * depression | 0.30 | 1.71/34.34 | .709 |

|  |  |  |  |  |
| --- | --- | --- | --- | --- |
| **Patients with / without relapse: N170 amplitude data** |  |  |  |  |
|  |  |  |  |  |
| \| **N170 go condition** \|  \| F \| Df \| Sig. \| \| --- \| --- \| --- \| --- \| --- \| \| Main effect \| group \| 0.17 \| 1/20 \| .679 \| \| n =11/12 \| depression \| 0.20 \| 1/20 \| .653 \| \|  \| electrode \| 0.23 \| 1/20 \| .631 \| \|  \| stimulus \| 0.14 \| 1.92/38.51 \| .860 \| \| Interactions \| group * electrode \| 1.42 \| 1/20 \| .247 \| \|  \| group * stimulus \| 1.41 \| 1.92/38.51 \| .256 \| \|  \| electrode * stimulus \| 0.88 \| 1.58/31.76 \| .399 \| \|  \| electrode * depression \| 0.93 \| 1/20 \| .345 \| \|  \| stimulus * depression \| 0.52 \| 1.96/38.51 \| .588 \| \|  \| group * electrode * stimulus \| 0.22 \| 1.58/31.76 \| .753 \| \|  \| electrode * stimulus * depression \| 0.26 \| 1.58/31.76 \| .722 \|  \| **N170 nogo condition** \|  \| F \| Df \| Sig. \| \| --- \| --- \| --- \| --- \| --- \| \| Main effect \| group \| 0.21 \| 1/20 \| .647 \| \| n =11/12 \| depression \| 0.93 \| 1/20 \| .346 \| \|  \| electrode \| 1.58 \| 1/20 \| .222 \| \|  \| stimulus \| 0.71 \| 1.88/37.59 \| .486 \| \| Interactions \| group * electrode \| 0.95 \| 1/20 \| .340 \| \|  \| group * stimulus \| 1.84 \| 1.88/37.59 \| .175 \| \|  \| electrode * stimulus \| 0.95 \| 1.38/27.60 \| .367 \| \|  \| electrode * depression \| 1.73 \| 1/20 \| .203 \| \|  \| stimulus * depression \| 0.63 \| 1.96/38.51 \| .525 \| \|  \| group * electrode * stimulus \| 0.20 \| 1.38/27.60 \| .736 \| \|  \| electrode * stimulus * depression \| 1.11 \| 1.38/27.60 \| .321 \| |  |  |  |  |
|  |  |  |  |  |
|  |  |  |  |  |
|  |  |  |  |  |
|  |  |  |  |  |
|  |  |  |  |  |
|  |  |  |  |  |
|  |  |  |  |  |
|  |  |  |  |  |
|  |  |  |  |  |
|  |  |  |  |  |
|  |  |  |  |  |
